# Surgical treatment of diffuse and multi-lobes involved glioma with the assistance of a multimodal technique

**DOI:** 10.1038/s41598-022-07287-0

**Published:** 2022-02-28

**Authors:** Jiayu Liu, Hewen Chen, Xin Gao, Meng Cui, Lin Ma, Xiaoque Zheng, Bing Guan, Xiaodong Ma

**Affiliations:** 1 Department of Neurosurgery, The First Medical Center, Chinese PLA General Hospital, Beijing, 100853 China; 2 Medical School of Chinese PLA, Beijing, China; 3 Department of Radiology, The First Medical Center, Chinese PLA General Hospital, Beijing, 100853 China; 4 Institute of Hospital Management, Medical Innovation Research Division of PLA General Hospital, Beijing, 100853 China

**Keywords:** Outcomes research, Surgical oncology, Neurological disorders

## Abstract

Diffuse and multi-lobes involved glioma (DMG) is a rare disease, and the aim of this study was to assess the role of multimodal-assisted surgical resection of tumours combined with chemoradiotherapy and identify prognosis. Clinical data were collected from 38 patients with a diagnosis of DMG. Nineteen patients received multimodal-assisted surgical resection of tumours combined with chemoradiotherapy, and another 19 patients underwent chemoradiotherapy alone after stereotactic puncture biopsy. The clinical characteristics, magnetic resonance imaging (MRI) findings, histopathological diagnosis, progression-free survival, and overall survival of DMG patients were retrospectively analysed. Twenty-six males and 12 females were included, and the age of the participants ranged from 10 to 80 years (46.34 ± 15.61). The median overall survival in our study was 25 months, and the progression-free survival was 17 months. The extent of resection was 50.10–73.60% (62.54% ± 7.92%). The preoperative and the postoperative KPS score of the patients in the operation group showed no statistically significant difference. The results of logistic regression demonstrated that overall survival was positively associated with operative treatment + chemoradiotherapy (p = 0.003) but negatively associated with age and corpus callosal involvement (p = 0.028 and 0.022, respectively). Kaplan–Meier analyses showed that those who underwent surgical treatment had a significant progression-free and overall survival benefit compared to those who did not undergo surgical treatment (log-rank test; p = 0.011 and 0.008, respectively). Older age and involvement of the corpus callosum represent a poor prognosis in DMG patients. Multimodal-assisted surgical resection of tumours combined with chemoradiotherapy might be a treatment option for DMG. Further research is needed to obtain the clear evidence of the effect of surgical treatment.

## Introduction

Diffuse and multi-lobes involved glioma (DMG)is a clinically rare primary diffuse invasive neoplastic lesion of the central nervous system. It was first named gliomatosis cerebri (GC) by Nevin in 1938^1^. However, it does not exist in the 2016 and 2021 World Health Organization (WHO) classifications It is characterized by diffuse neoplastic proliferation of glial cells in the brain parenchyma while the original anatomical structure of the tissue remains relatively intact; the proliferation invades more than 3 lobes, often bilateral, and often extends to the brainstem and thalamus. Biopsy pathological examination of this disease should be combined with clinical imaging to make the final diagnosis^2^. However, the degree of tumour invasion is not related to the histology and clinical symptoms of the tumour^1^. Although GC is no longer used in the last two WHO classification edition, the disease is viewed as a growth model in a variety of diffuse gliomas^3^. In addition, this term was used widely until recently^4^. In order to avoid misunderstanding by using terms that no longer exist, we used DMG instead of GC in this study.

Moreover, patients with DMG generally have a poorer prognosis than patients with the corresponding grade of diffuse glioma^5^. This prognosis may be associated with widespread DMG in multiple lobes of the brain, and treatment options are limited. Due to the wide range of tumours and unclear boundaries, surgery is difficult to complete, so biopsy is currently not advocated for surgical resection and is only a means of diagnosis^6^. Currently, radiotherapy is the main method used to treat DMG and can significantly prolong the survival time of most patients. Some studies also suggest that early chemotherapy has certain efficacy in controlling DMG progression^7,8^.

However, with the development of the neuronavigational system, the combination of multimodal imaging, neuronavigational systems and intraoperative magnetic resonance imaging (iMRI) can more accurately determine the tumour boundary, improve the degree of total tumour resection, better protect brain function, greatly reduce the risk of surgery and be an indication of decision making process in leaving tumour behind to prevent complication^9,10^. The benefits of iMRI include its display of real-time progress of lesion resection and provision of crucial reference information that enables the surgeon to create an effective operation plan. The advantage is critical when a lesion is adjacent to important structures, such as the pyramidal tract^11^. The method can also rebuild images of important nerve structures, such as white matter fibers, and update the operation plan continually throughout the procedure, thus helping surgeons adjust to intracranial tissue shifts during operation and allowing for the modification of white matter fiber bundle reconstruction before the procedure^12^. In this study, the clinical characteristics, imaging findings, histopathological diagnosis, progression-free survival, and overall survival of DMG patients were retrospectively analysed. The clinical efficacy of multimodal-assisted surgical resection of tumours combined with chemoradiotherapy and chemoradiotherapy alone were compared to provide guidance for future clinical diagnosis and selection of appropriate treatment.

## Results

### Baseline characteristics

A total of 38 patients (12 males; 26 females) were included, and the age of the participants ranged from 10 to 80 years (46.34 ± 15.61); among these, 19 patients underwent multimodal-assisted surgical resection of tumours combined with chemoradiotherapy, and the other 19 patients underwent chemoradiotherapy alone. The duration of disease ranged from 1 to 270 days (46.55 ± 53.85), and the preoperative KPS ranged from 30 to 100. It is not standard practice to offer surgery to individuals with KPS < 30. In this study, there was only 1 patient with KPS < 30. We performed operation on this patient in order to save his life. Postoperative KPS ranged from 10 to 100. The presenting symptoms were focal sensorimotor deficits for 20 patients (52.60%), seizures for 11 patients (28.90%), increased intracranial pressure (ICP) (headache, nausea/vomiting) for 13 patients (34.20%), and cognitive deficits for 11 patients (26.30%). Signalling patterns on MRI generally showed T1-weighted hypointensity and T2-weighted hyperintensity. Radiographic findings including the lobes and/or distinct brain regions involved ranged from 3 to 10 (4.82 ± 2.04). Most patients (65.80%) had bilateral involvement. Seventeen patients (44.70%) had corpus callosal involvement, 20 patients (52.60%) had thalamic involvement, and 13 patients (34.20%) had brainstem involvement. According to preoperative imaging results, the mean tumour volume was 85.73 ± 35.65 cm^3^ (ranged from 50.5 to 160.9). In the operation group, the mean postoperative tumour volume was 51.17 ± 14.91 cm^3^. The extent of resection was 50.10–73.60% (62.54% ± 7.92%). There was no correlation between extent of resection and KPS (p = 0.990).

Histology was available in all 38 cases. Grade II glioma was diagnosed in 18 patients (47.40%), grade III in 10 patients (26.30%), and grade IV in 10 patients (26.30%). In other patients, the pathological diagnosis was Diffuse astrocytoma in 6 patients (15.80%), Oligodendroglioma in 3 patients (7.90%), Oligoastrocytoma in 3 patients (7.90%), Anaplastic astrocytoma in 3 patients (7.90%), Anaplastic oligodendroglioma in 4 patients (10.50%), Glioblastoma in 9 patients (23.70%) and others (astrocytoma in 6 patients, gemistocytic astrocytoma in 2 patients, diffuse midline glioma in 1 patients, and giant cell glioblastoma in 1 patients). MGMT promoter methylation was found in 26 patients (68.4%). Immunohistochemical analysis for IDH1 mutation was found in 4 patients (10.50%). Loss of ATRX expression was observed in 17 patients (44.70%). Positive nuclei of Olig-2 were found in 33 patients (86.80%), and high expression of GFAP was found in 36 (94.70%). Nuclear expression of p53 was detected by immunohistochemistry in 34 patients (89.50%). One patient (2.63%) with midline extension showed an H3-K27 M mutation. Ki‑67 protein expression was 2–80% (27.76% ± 22.58%). There was no significant difference between the operation group and the non-operation group in terms of baseline characteristics. (Table 1).

**Table 1:** Clinical characteristics of groups.

Factor	Total	Operation group	Non-operation group	p value
Age-year	46.34 ± 15.61	47.00 ± 13.28	45.68 ± 17.99	0.799
**Gender**				0.484
Male sex—no. (%)	26 (68.42%)	14 (73.68%)	12 (63.16%)	
Female sex—no. (%)	12 (31.58%)	5 (26.32%)	7 (36.84%)	
Duration of disease—day	46.55 ± 53.85	50.47 ± 48.09	42.63 ± 60.13	0.660
**Preoperative KPS (%)**				0.447
80–100	16	9	7	
50–70	19	9	10	
0–40	3	1	2	
**Postoperative KPS (%)**				0.197
80–100	17	10	7	
50–70	16	7	9	
0–40	5	2	3	
**Presenting neurologic symptom**				
Focal sensorimotor deficit—no. (%)	20 (52.60%)	9 (47.37%)	11 (57.89%)	0.515
Seizure—no. (%)	11 (28.90%)	7 (36.84%)	4 (21.05%)	0.281
Increased ICP (Headache, vomiting)—no. (%)	13 (34.20%)	4 (21.05%)	9 (47.37%)	0.084
Cognitive deficits—no. (%)	11 (26.30%)	4 (21.05%)	6 (31.58%)	0.460
Lobes/cortical areas involved	4.82 ± 2.04	4.74 ± 2.08	4.89 ± 2.05	0.815
**Sides**				0.359
Bilateral involvement—no. (%)	25 (65.80%)	12 (63.16%)	13 (68.42%)	
Only left hemispheric involvement—no. (%)	7 (18.40%)	2 (10.53%)	1 (5.26%)	
Only right hemispheric involvement—no. (%)	6 (15.80%)	5 (26.32%)	2 (10.53%)	
Corpus callosal involvement—no. (%)	17 (44.70%)	9 (47.37%)	8 (42.11%)	0.744
Brainstem involvement—no. (%)	13 (34.20%)	4 (21.05%)	9 (47.37%)	0.084
Thalamic involvement—no. (%)	20 (52.60%)	9 (47.37%)	11 (57.89%)	0.515
**Histologic grade**				1.000
Grade 2—no. (%)	18 (47.40%)	9 (47.37%)	9 (47.37%)	
Grade 3—no. (%)	10 (26.30%)	5 (26.32%)	5 (26.32%)	
Grade 4—no. (%)	10 (26.30%)	5 (26.32%)	5 (26.32%)	
**Histopathology**				0.193
Diffuse astrocytoma—no. (%)	6 (15.80%)	4 (21.05%)	2 (10.53%)	
Oligodendroglioma—no. (%)	3 (7.90%)	1 (5.26%)	2 (10.53%)	
Oligoastrocytoma—no. (%)	3 (7.90%)	1 (5.26%)	2 (10.53%)	
Anaplastic astrocytoma—no. (%)	3 (7.90%)	1 (5.26%)	2 (10.53%)	
Anaplastic oligodendroglioma—no. (%)	4 (10.50%)	4 (21.05%)	0	
Glioblastoma—no. (%)	9 (23.70%)	4 (21.05%)	5 (26.32%)	
astrocytoma—no. (%)	6 (15.80%)	5 (26.32%)	1 (5.26%)	
Gemistocytic astrocytoma—no. (%)	2 (5.26%)	0	2 (10.53%)	
Diffuse midline glioma—no. (%)	1 (2.63%)	1 (5.26%)	0	
Giant cell glioblastoma—no. (%)	1 (2.63%)	0	1 (5.26%)	
MGMT promoter methylation—no. (%)	26 (68.4%)	16 (84.21%)	10 (52.63%)	0.081
IDH-1 mutation—no. (%)	4 (10.50%)	3 (15.79%)	1 (5.26%)	0.281
Loss of ATRX expression—no. (%)	17 (44.70%)	11 (57.89%)	6 (31.58%)	0.101
OLIG-2—no. (%)	33 (86.80%)	18 (94.74%)	15 (78.95)	0.138
GFAP—no. (%)	36 (94.70%)	19 (100.00%)	17 (89.47%)	0.089
P53 IHC—no. (%)	34 (89.50%)	18 (94.74%)	16 (84.21%)	0.281
H3K27M IHC—no. (%)	1 (2.63%)	1 (5.26%)	0	0.311
Ki-67	27.76 ± 22.58	25.37 ± 23.29	30.16 ± 22.21	0.521

### Overall survival is associated with age, corpus callosal involvement and treatment method

**Figure 1:**
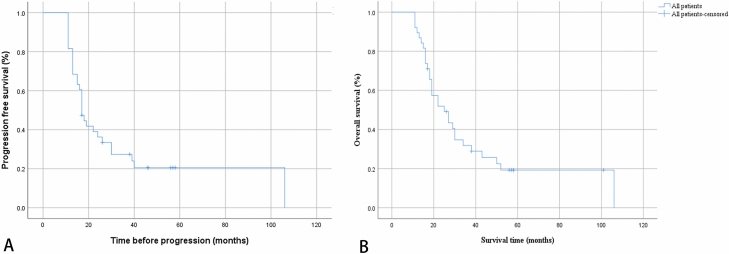
Survival Analysis in Total. Data were available in all 38 patients. Kaplan–Meier analyses: (**A**) Median progression-free survival was 17 months. (**B**) Median overall survival was 25 months.

Survival data were available in all 38 patients. The median overall survival in our study was 25 months, and the progression-free survival was 17 months. (Fig. 1) We divided 38 patients into longer-than-the-median overall survival and shorter-than-the-median overall survival. Logistic regression was used to assess baseline data, MRI and pathology findings and treatment methods relative to overall survival. The results demonstrated that overall survival was positively associated with operative treatment + chemoradiotherapy (p = 0.003) but was negatively associated with age and corpus callosal involvement (p = 0.028 and 0.022, respectively) (Table 2). We did not find the prognostic relevance of molecular features correlation with overall survival, MGMT promoter methylation (p = 0.494), IDH-1 mutation (p = 0.577), loss of ATRX expression (p = 0.208), OLIG-2 (p = 0.576), GFAP (p = 0.531), P53 IHC (p = 0.682), H3K27M IHC (p = 0.737) and Ki-67 (p = 0.920). In logistic regression analysis, the histological grade did not affect the OS (p = 0.156).

**Table 2:** Logistic regression analysis of factors associated with overall survival.

Factor	B	S.E	DF	Sig	EXP(B)	95% CI of EXP (B)	
						Minimum	Upper limit
Age	− 0.087	0.039	1	0.028	0.917	0.849	0.991
Corpus callosal involvement	− 2.950	1.289	1	0.022	0.052	0.004	0.654
Operation treatment	3.714	1.266	1	0.003	0.024	0.002	0.292

*B* regression coefficient, *S.E*. standard error, *Sig*. significance, *df* degree of freedom, *exp* odd ratio.

### Treatment methods and survival

The preoperative and postoperative KPS score of the patients in the operation group showed no statistically significant difference (p = 0.863). The preoperative and postoperative KPS score of the patients in the non-operation group also showed no statistically significant difference (p = 0.135).

**Figure 2:**
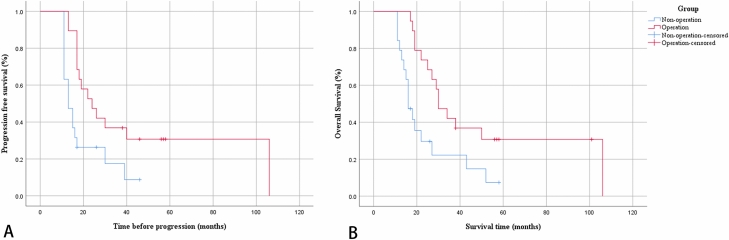
Survival analysis between groups. Kaplan–Meier analyses: (**A**) the progression-free survival was 24 months in the comprehensive treatment group and 13 months in the nonoperation group. Patients who received comprehensive treatment had a significant progression-free survival benefit compared to those who did not receive comprehensive treatment (log-rank test, p = 0.011). (**B**) The overall survival was 30 months in the comprehensive treatment group and 16 months in the nonoperation group. Patients who received comprehensive treatment had a significant overall survival benefit compared to those who did not receive comprehensive treatment (log-rank test, p = 0.008).

The progression-free survival was 24 months in the operation group, and the overall survival was 30 months. The progression-free survival was 13 months in the non-operation group, and the overall survival was 16 months. Treatment methods correlated with progression-free survival and overall survival. Patients who received surgical treatment had a significant survival benefit compared to those who did not receive surgical treatment (log-rank test; p = 0.011 and 0.008, respectively) (Fig. 2).

## Discussion

The incidence of DMG is rare, estimated at 1.5 cases per 10 million in the population^13^. Therefore, its histopathological phenotype, genotype and clinical treatment strategy are still unclear^5^. In this study, DMG can occur in children to the elderly (10–80 years old) but mostly in middle-aged people, with an average age of 46.34 ± 15.61 years old, and is more common in men. The DMG onset for most DMG patients is slow and insidious, and the longest duration of DMG patients can reach up to 270 days. The clinical manifestations are complex and varied, and there is a lack of characteristic manifestations in the early stage of the disease. The main clinical symptoms were focal sensorimotor deficits (52.60%) and seizures, increased ICP and cognitive deficits. These observations are consistent with those of previous studies^14,15^. In this study, MRI examination of the patients showed T1-weighted hypointensity and T2-weighted hyperintensity in multiple lobes that involved the corpus callosum, thalamus and even the brainstem. Most of the patients in this study had bilateral lesion involvement. The boundaries between the involved lesions and normal brain tissue were not clear, and there was no obvious space occupation or enhancement. Histopathological analysis confirmed that the histological grade of glioma was WHO grade II to IV, with grade II being the most common. With the development of molecular pathology, an increasing number of molecular markers can help us to determine the prognosis of DMG more accurately. A study^16^ reported that IDH1 mutation in adult DMG reaches 17–84%, but the mutation rate in this study was lower than this level. Due to the small number of cases in this study and the main purpose of this study is the effect of surgery, it is difficult to explain the reasons for this phenomenon, and further analysis is necessary. In addition, IDH1 mutation and MGMT methylation might be prognostic factors for DMG^17^. Molecular markers such as IDH1, ATRX and H3K27 were investigated in this study, but no conclusive results of specific molecules were found, and no prognostic effect was found. This result may be due to the insufficient sample size of this study and the fact that patients in this study received two different treatments. To date, no studies have found that the molecular study of DMG is unique compared with other gliomas^18,19^. Therefore, further studies are needed to demonstrate the prognostic role of molecular markers.

The median overall survival in our study was 25 months, and the progression-free survival was 17 months. (Fig. 1). This result is similar to that of previous studies^7,20,21^. We divided 38 patients into longer-than-the-median overall survival and shorter-than-the-median overall survival. The results of logistic regression demonstrated that overall survival was positively associated with operative treatment + chemoradiotherapy but was negatively associated with age and corpus callosal involvement (p < 0.05). The influence of age on the prognosis of glioma has also been recognized by most scholars^22^. The influence of age on prognosis is attributed to the fact that elderly patients have poorer tolerance to surgery, radiotherapy and chemotherapy and have more aggressive tumours than young people^23^. Corpus callosum invasion has been identified as a factor contributing to poor prognosis in glioma patients^24^, and this study also found this feature in DMG patients. The corpus callosum is a combination of large white matter connecting the two hemispheres of the brain, which is an important way for glioma of the corpus callosum to spread to the hemispheres of the brain or for involvement of one hemisphere of the brain to spread to the opposite hemisphere of the brain^25^. Gliomas involving the corpus callosum are often located deep in the brain. It is worth mentioning that this study did not show a correlation between WHO classification and prognosis, which is consistent with a previous DMG study^26^. Although the pathology of DMG may be grade WHO II, III, or IV, the prognosis is generally poor because of the wide range of lesions involved^5^. In addition, we did not find the prognostic relevance of thalamic and brainstem involvement correlation with overall survival (p > 0.05). However, thalamic and brainstem involvement is an important factor affecting GC outcomes in clinical practice. In this study, only 38 patients were divided in two groups, so we do not have enough data to make a stratified comparison by lobes involvement. However, this is a valuable consideration for a future study.

Currently, treatment of DMG is still in the exploratory stage, and controversy exists regarding treatment of the disease. The main treatment methods include surgery, chemotherapy and radiation therapy. Our logistic regression results demonstrated that overall survival was positively associated with operative treatment + chemoradiotherapy. When patients were divided into the operation and nonoperation groups, there was a trend towards increased progression-free survival and overall survival in the operation group. Current studies suggest that radiation therapy is an effective method for treatment of brain gliomatosis and can significantly prolong the survival time of patients^27,28^. The cyber knife is an effective treatment modality for glioma^29^. It has also been reported that early chemotherapy has a certain effect on controlling the progression of brain gliomatosis^7^. Kong et al.^30^ conducted a retrospective study on 37 DMG patients and found that the survival time of patients who received radiotherapy-assisted chemotherapy was significantly longer than that of patients who received radiotherapy alone. In our study, the progression-free survival was 13 months in the nonoperation group that underwent chemoradiotherapy alone, and the overall survival was 16 months, which is similar to Kong’s study^30^.

Due to the wide range of DMG lesions, it is difficult to completely remove, so surgical treatment is not recommended in this study. To clarify the pathology, biopsy of intracranial lesions can be performed under stereotactic or neuronavigation-guided surgery^31^. However, we believe that tumour resection should be maximized under the principle of preserving brain function as much as possible to achieve the objective of clarifying pathology, relieving high cranial pressure, reducing tumour volume, and improving the efficacy of chemoradiotherapy. The classical STUPP protocol consists of: a combination maximal surgical resection, followed by concurrent radiotherapy and chemotherapy with TMZ^32^. It remains the standard treatment for a newly diagnosed Glioblastoma^33^. In this study, we treated DMG patients with this protocol. And in addition to the classical STUPP protocol, we also used the cyber knife treatment. Perez-bovet et al.^34^ performed temporal lobectomy to reduce high intracranial pressure in a patient, followed by radiotherapy and chemotherapy to prolong the patient's life for 23 months. Chen et al.^27^ proposed that surgery could improve the efficacy of radiotherapy for patients, thus improving the quality of life and prolonging the survival time. The primary goal of glioma surgery is to maximize the removal of the tumour while minimizing the trauma, thus improving the patient's quality of life. Neurosurgeons usually need to consider the relationship between anatomic and functional modes of a certain site during tumour resection. Multimodal technology can combine two or more modes so that the multiple modal information of the brain can be collected in an information system, which can be used as a tool for the operator to evaluate spatial and functional information^35^. Compared with traditional and neuronavigational microsurgery, iMRI-based multimode techniques can achieve a higher total resection rate^36^. Because DMG involves multiple lobes, operation is highly likely to result in limb paralysis and/or other neurological disorders. Multimodal techniques, including DTI, fMRI, MRA, MRV and MRS, can be fully applied in intraoperative neural function navigation, thus significantly improving the degree of tumour resection while preserving normal neural function^37^.

To the best of our knowledge, this is the first study of surgical treatment for DMG with the assistance of a multimodal technique. Because of the wide range of gliomatosis in the brain, it is difficult to remove completely. In our study, the extent of resection was 50.10–73.60% (62.53 ± 7.92%) in the operation group. Patients with a higher degree of tumour resection are more likely to have better survival^38^. Our results suggest that for patients with DMG, surgical resection of approximately 60% may prolong patient survival. And it is not easy to quantify the extent of surgical resection as a simple percentage. Several variables must be considered here, including involvement of the dominant or non-dominant hemisphere, involvement of eloquent cortical areas, and the involvement of critical intracranial structures such as the brainstem. However, due to the limited sample size of patients treated by surgery in this study, further studies are needed to prove this conclusion. In addition, maximum tumour resection can greatly reduce the tumour volume in the local area, and at the same time, tumour cells resistant to radiation therapy can be removed, increasing radiotherapy dose per unit volume of residual mass^39^. Furthermore, a large degree of tumour resection can greatly reduce intracranial pressure, improve the tolerance to chemoradiotherapy, reduce clinical symptoms, and improve the quality of life for patients. Especially for high-grade patients who contrast-enhanced part of the infiltrating tumor was measured, the part of the tumour that is high grade was removed and probably most affects the OS. In this study, the progression-free survival and overall survival of patients in the operation group were significantly better than those in the nonoperation group (24 vs. 13 and 30 vs. 16 months, respectively). Moreover, the survival time of the operation group was longer than that in previous DMG-related studies^7,8,40,41^. There was no statistically significant difference in preoperative and postoperative KPS scores, which proved that the surgery did not cause a decrease in patient quality of life. Considering that the study's sample size is relatively small, further research is necessary to get more accurate conclusion. It is worth mentioning that although there was no significant difference between surgery group and biopsy group in terms of MGMT promoter methylation which is associated with a better prognosis, the proportion of MGMT promoter methylation in the operation group was still higher than that in the non-operation group (84.21% vs. 52.63%). It is possible that this was the factor associated with improved prognosis in the patients versus the actual intervention of surgery + chemoradiotherapy.

Some limitations must be considered in our study. First, this study was limited by an insufficient sample size. This disease is extremely rare, so it is difficult to obtain a sufficiently large number of cases for more statistically reliable results. In addition, the clinical data of early patients cannot meet the new WHO Brain tumor classification and not carried out relevant molecular analysis (such as codeletion 1p19q). The limitation of the study is molecular analysis, so the prognostic relevance of molecular features still needs further study. Second, our study was a retrospective study, and a randomized controlled trial should be performed in the future. Third, the extent of surgical resection that provides a survival benefit is still unclear. Although the extent of resection was 50.10–73.60% (62.53 ± 7.92%) in the operation group, this result does not mean that it is enough when the tumour is involving 3 cerebral lobes. Further multicenter large sample studies are necessary to determine the tumor resection threshold for patient’s benefit. Finally, the iMRI system can provide neurosurgeons with objective evidence of the extent of lesion resection. However, the tumour boundaries shown by MRI images do not fully represent the biological boundaries of tumour cells. Further research is needed to determine the biological boundaries of tumours by MRI.

## Conclusion

The incidence of DMG is very low. To the best of our knowledge, this study is the first to analyse multimodal-assisted surgical treatment of DMG. Our findings suggest that older age and involvement of the corpus callosum represent a poor prognosis in DMG patients. In addition, our study supports the general employment of surgery with the assistance of multimodal techniques in management of DMG patients beyond biopsy to obtain a histologic diagnosis. However, there is no clear evidence that surgery in addition to chemoradiotherapy includes outcomes, especially in a study with a small sample size. In conclusion, our study shows that multimodal-assisted surgical resection of tumours combined with chemoradiotherapy might be a treatment option for DMG. Further research is needed to obtain the clear evidence of the effect of surgical treatment.

## Methods

### Patients

Clinical data from 38 patients with a diagnosis of DMG were collected between January 2007 and January 2019 at the Department of Neurosurgery, the First Medical Center of PLA General Hospital. We retrospectively analysed the data of the above patients. Only patients with magnetic resonance imaging (MRI) illustrating involvement of at least three cerebral lobes and histology consistent with diffuse glioma were included for further study^27^. All cases were of primary glioma, in which there was no pre-existing focal glioma prior to initial presentation^5^. Written informed consent was obtained from each participant, and the study was approved by the institutional review board of the hospital. (The full name of the ethics committee: the institutional review board of the First Medical Center of PLA General Hospital) All methods were performed in accordance with the relevant guidelines and regulations.

Baseline data and medical history were obtained from patient medical records. Baseline data included age, sex, course of disease, presenting neurologic symptoms, imaging findings, and histopathological diagnosis. MRI was conducted every three months after surgery. Survival in the study refers to mortality. Progression-free survival was defined as the time from initial surgery and demonstration of an increase in tumour size on follow-up MRI. Preoperative and postoperative status was based on the Karnofsky performance score. The functional status of a patient was assessed on an 11-point scale ranging from full well-being (100%) to death (0%), decreasing by ten points at each level^42^.

### Magnetic resonance imaging

1.5 T intraoperative magnetic resonance imaging (iMRI) examination (Siemens Espree, Erlangen, Germany) was performed, including 3D T1 weighted and postcontrast 3D T1-weighted magnetization prepared rapid-acquisition gradient echo sequences, T2- weighted sequences, 3D T2 fluid-attenuated inversion recovery sequences, diffusion tensor imaging (DTI), functional magnetic resonance imaging (fMRI), magnetic resonance angiography (MRA), magnetic resonance angiography (MRV), and magnetic resonance spectroscopy (MRS). The image processing was consistent with a previous study from our team^43^.

**Figure 3:**
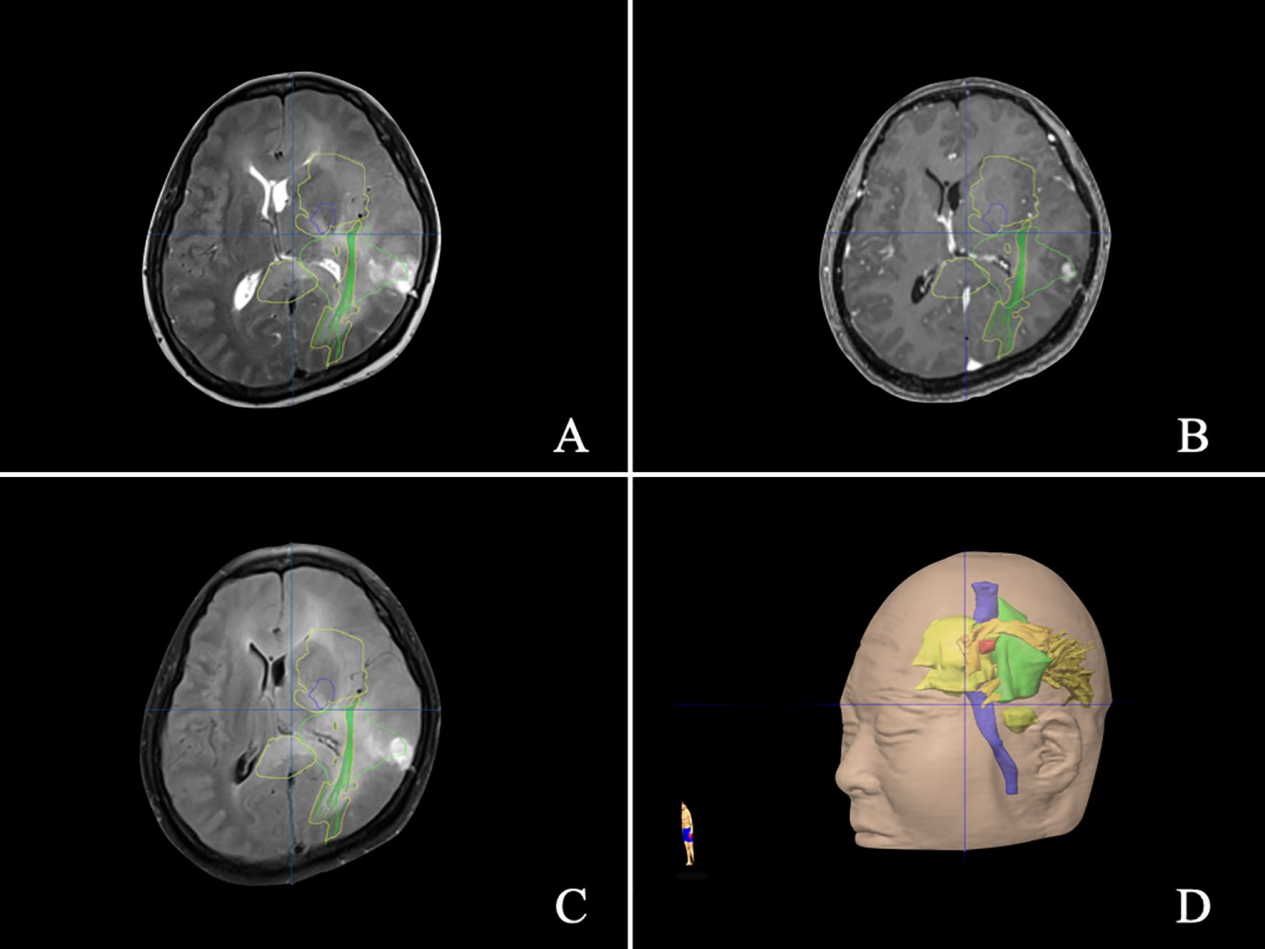
Illustration of the multimodal technique in Case 1. Preoperative MRI and multimodal reconstruction of a 41-year-old man with diffuse and multi-lobes involved glioma. A preoperative axial (**A**–**C**) magnetic resonance imaging (MRI) scan showed a giant glioma. (**D**) Three-dimensional profile of the tumour. The arcuate tract is shown in brown. The optical radiation is shown in yellow. The pyramidal tract is shown in purple. The Broca area is shown in red. A tumour is shown in green.

In brief, based on the 3D T1-weighted sequences, 3D T2-weighted sequences, DTI, fMRI, MRA, MRV, and MRS data, a multimodal operation plan was developed using the neuronavigation planning software iPlan 2.6 (BrainLab, Feldkirchen, Germany) (Fig. 3). T1-weighted enhanced sequencing was used for 3D reconstruction of high-grade tumours, and FLAIR sequencing was used for low-grade tumours. DTI mode was used for tracer reconstruction of white matter fibre bundles. fMRI mode was used for tracer reconstruction of the visual cortex, language area and other important functional areas; MRA mode was used for reconstruction of important arteries in the surgical area; MRV mode was used for reconstruction of important veins in the surgical area; and MRS mode was used for reconstruction of the tumour metabolic area.

### Surgical treatment with the assistance of a multimodal technique

**Figure 4:**
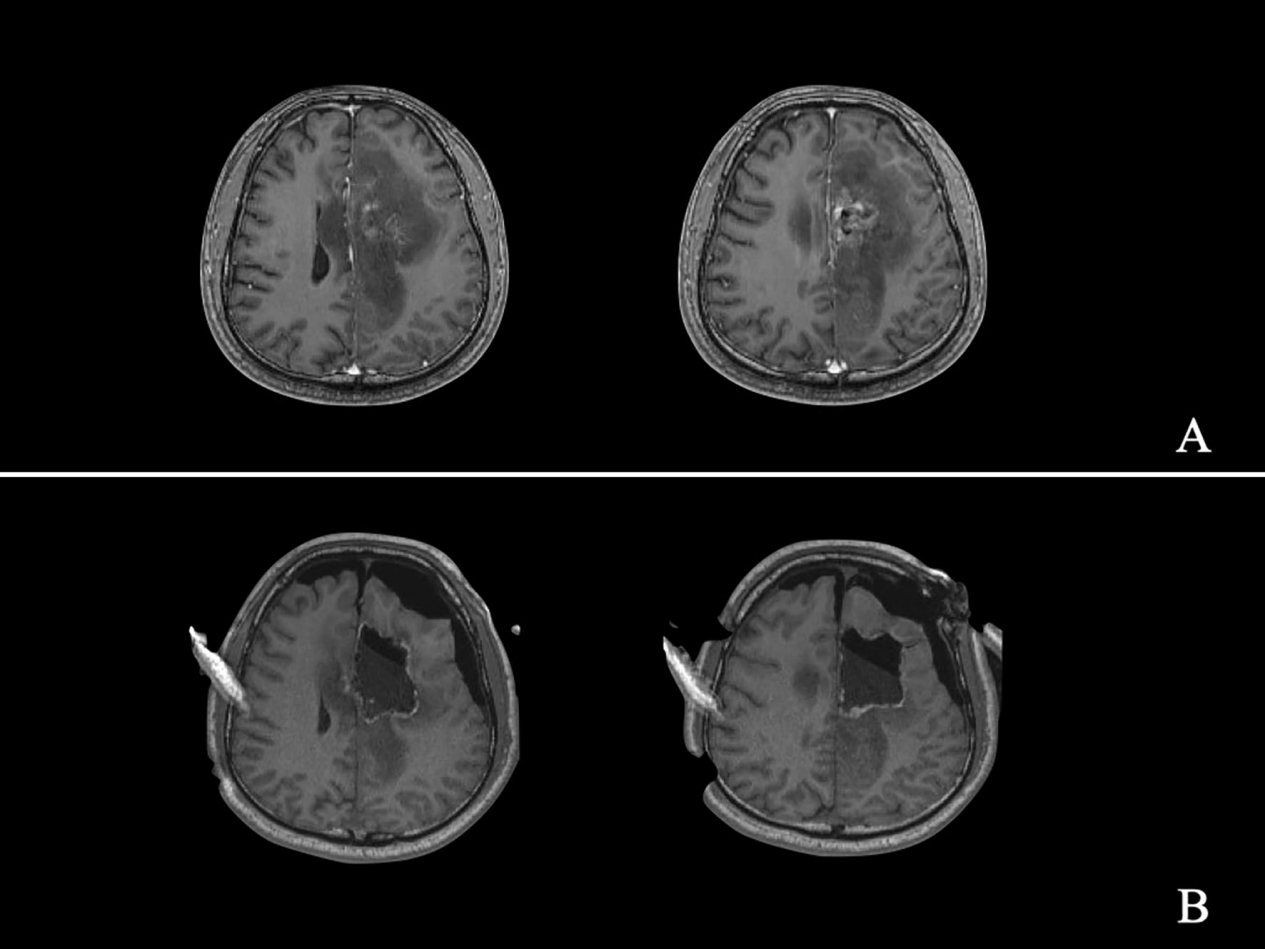
Illustrative Case 2-a. A 33-year-old man was diagnosed with diffuse and multi-lobes involved glioma, which was successfully partially removed. Preoperative axial (**A**) MRI scan showed a giant tumour. An intraoperative axial (**B**) MRI scan showed partial tumour removal.

**Figure 5:**
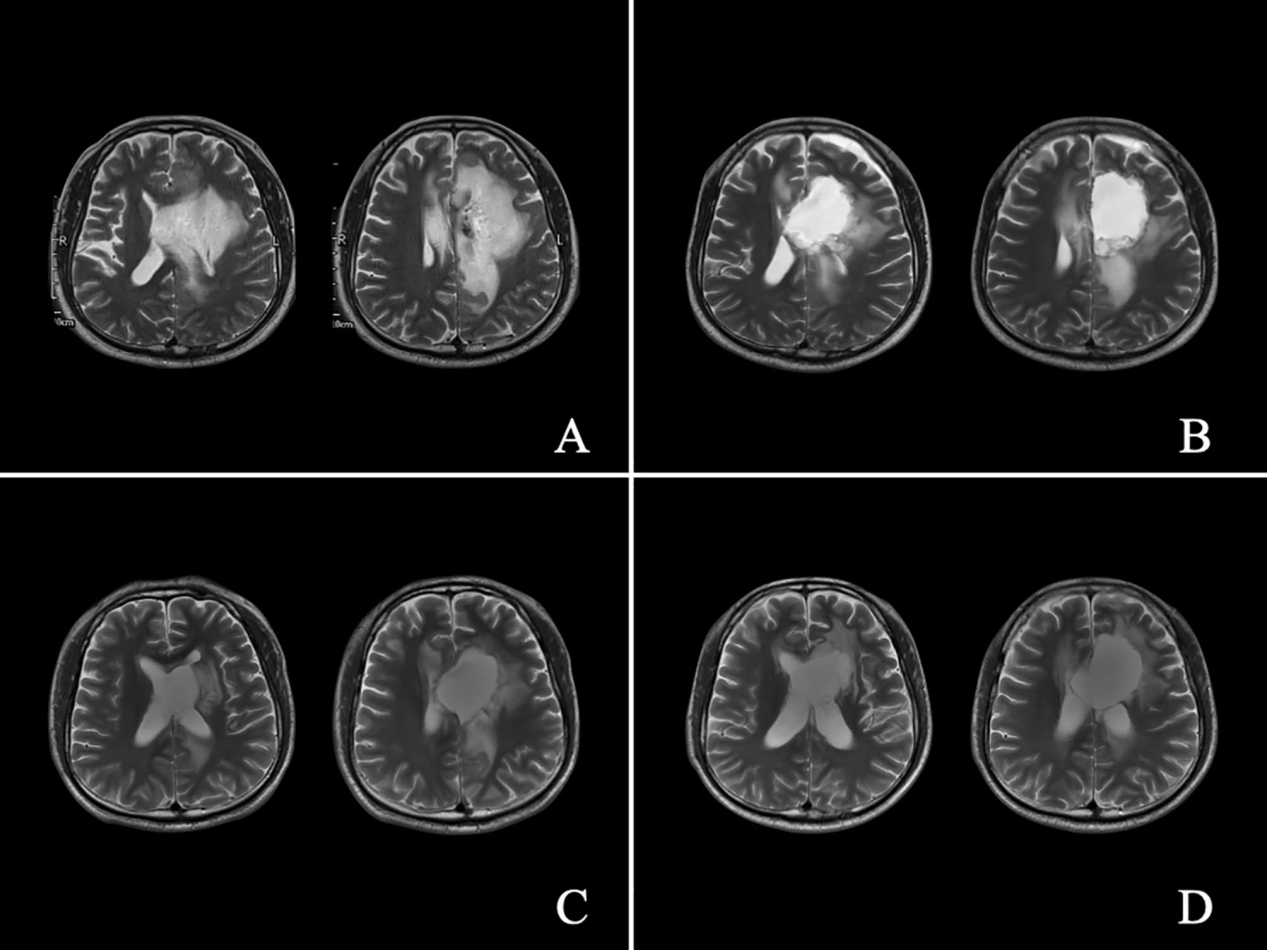
Illustrative Case 2-b. Preoperative axial (**A**) T2-weighted MRI scan; axial (**B**) MRI scan before discharge after surgery; axial (**C**) MRI scan at 12 months after surgery; axial (**D**) MRI scan at 41 months after surgery.

The neurosurgeon communicated with the patients and/or families of patients preoperatively. Treatment options were selected based on the wishes of patients and/or families of patients after adequate preoperative evaluations. Nineteen patients received surgical treatment. The surgery was performed under general anaesthesia. A neuronavigation microscope (Pentero; CarlZeiss, Oberkochen, Germany) could display the contour of the tumour and the functional data during surgery^35^. All of the processed image data were integrated for surgical planning and intraoperative neuronavigation. The cortical language area, arcuate fasciculus, and pyramidal tract were identified using functional neuronavigation. Gliomatosis has a wide range of invasions and aggressive growth, and it was difficult to achieve complete radical resection. The purpose of the operation was palliative treatment with maximum excision of the lesion (Figs. 4, 5). The surgeon considered that tumour resection had been achieved, the surgery was suspended, and iMRI scanning was performed to quickly evaluate the surgical results. In addition, intraoperative electrophysiological monitoring guaranteed functional protection. The surgical process was monitored by an electrophysiologist who warned if there were abnormalities in motor evoked potential, somatosensory evoked potential, or visual evoked potential. The tumour volume of pre- and postoperative MRI was analysed by the same neurosurgeon. Manual segmentation was performed with region-of-interest analysis to measure the tumour volumes (cm^3^) based on contrast-enhancing tissue observed on postcontrast T1-weighted MR images or T2 fluid-attenuated inversion recovery sequence images^35^. Contrast-enhanced part of the infiltrating tumor was measured in high-grade patients. The general extent of resection for treating GC is to first attempt as complete an excision of tumor as possible (based on postsurgical MRI verification) without compromising function. However, for tumors that involve thalamic/brainstem, a total removal may not be feasible, and an aggressive approach could result in neurologic deficits^44^.

In contrast, another 19 patients underwent stereotactic puncture biopsy. The biopsy procedure was performed according to the instructions of the VarioGuide system and software (VarioGuide; BrainLAB, Heimstetten, Germany). iMRI was performed when the planned targets were thought to be achieved. Details of trajectory planning are described in our previous reports^45^. Histological diagnosis was diagnosed according to the 2016 WHO classification^3^. Immunohistochemical analyses were performed using antibodies against Ki-67, GFAP, Olig-2, ATRX, p53, IDH1 (R132H), and histone H3K27 M mutations in tumours involving the midline^46^.

#### Chemoradiotherapy

**Figure 6:**
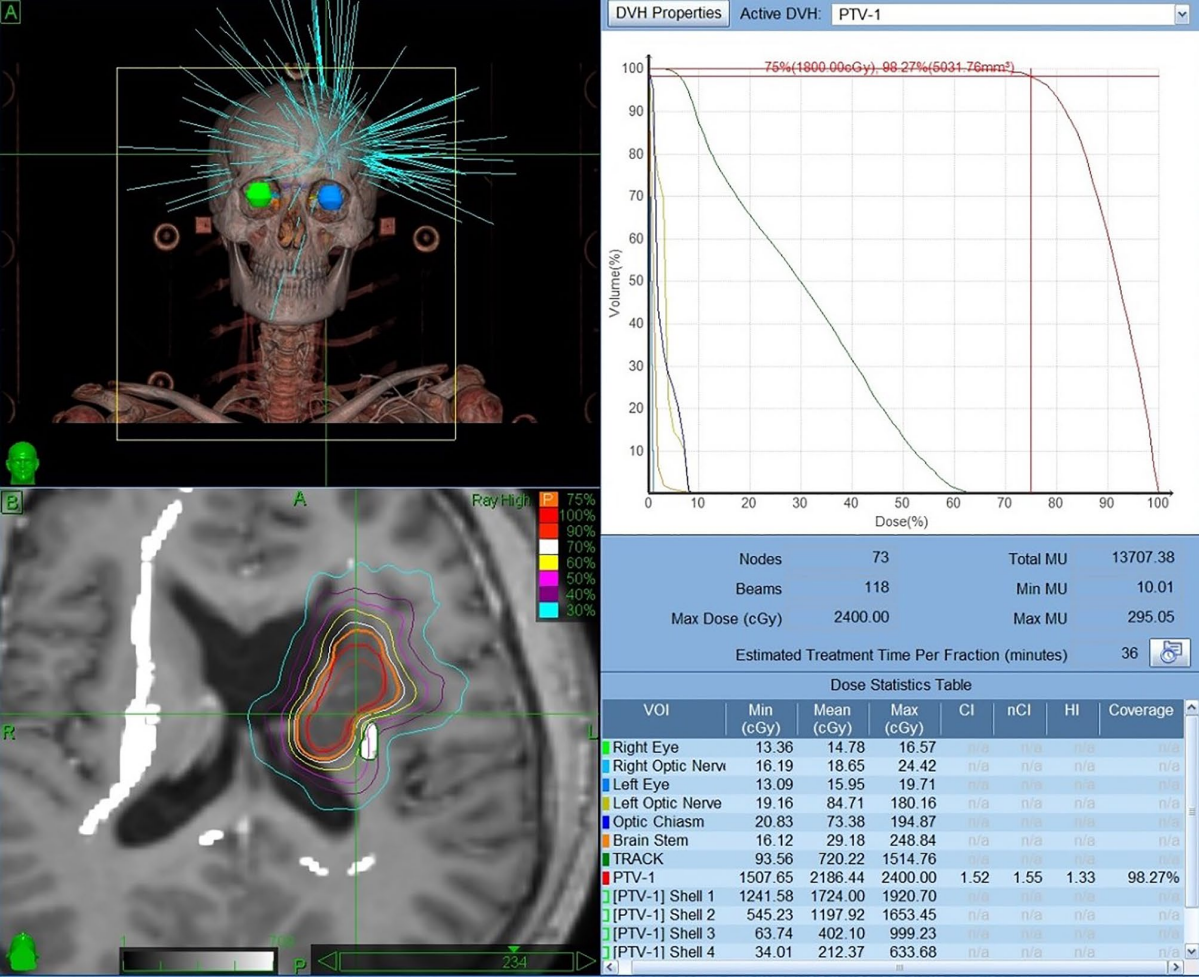
Illustrative Case 2-c. This patient received 2400 cGy CyberKnife therapy at 6 months after surgery.

The strategy of radiotherapy and chemotherapy combined with surgery in this study was based on the guideline in 2005^47^, and the treatment regimen is also reasonable according to the NCCN guidelines, Central Nervous System Cancers (Version 3.2020)^32,44^. Low grade patients were on radiotherapy + adjuvant temozolomide and high-grade patients were on chemoradiotherapy plus adjuvant 6 cycles of temozolomide (Stupp protocol) after surgery or biopsy^44^. For low-grade gliomas, we did not use concomitant radiotherapy, and for high-grade gliomas, we used concomitant radiotherapy as per the Stupp protocol. The gross tumour volume (GTV) was defined on the basis of the T2-weighted MRI and FLAIR sequence MRI. The clinical target volume (CTV) included GTV edge extension of 1–2 cm. A radiotherapy dose of 1.8–2 Gy/day was delivered, with a total dose of 40–60 Gy. Patients received temozolomide (TMZ) at 75 mg/m^2^ daily throughout radiotherapy followed by adjuvant 6 cycles of TMZ 150–200 mg/m^2^ (days 1–5) every 4 weeks after irradiation. In addition, all of the patients underwent functional neuroimaging as well as anatomical MRI and CT for CyberKnife radiosurgery treatment plans. The Cyber knife is not usually used in the first line of treatment of diffuse glial CNS tumor. In our study, patients received the Cyber knife boost following chemoradiation. (Fig. 6) The GTV was defined, on the basis of anatomical MRI sequences, as the contrast-enhancing area. A second volume indicated as a planned treatment volume (PTV) was defined as GTV edge extension of 0.2–0.5 mm. The median prescription dose to the tumour was 21 Gy (range 14–22.5 Gy), delivered in a median of 3 fractions (range: 1–3 fractions).

### Statistical analysis

SPSS statistical software 19.0 (IBM Corp., Armonk, NY, USA) was used for data analysis. Numerical variables are expressed as the mean ± SD. Qualitative variables are described as the absolute value of cases in the distinctive group. Statistical significance between the quantitative variables was assessed with the χ^2^ test and with Yates's or Fisher's correction if necessary. Student's t test was performed to evaluate the data with a normal distribution. Repeated measure analysis of variance was used for statistical assessment. The association between the indices was statistically analysed using logistic regression. The survival data of different groups were determined using the Kaplan–Meier method and compared using the log-rank test^48^. Univariable and multivariable Cox proportional hazards regression models were used to estimate the association of exclusion criteria and other clinically relevant prognostic factors. Pearson correlation coefficient was made between extent of resection and Karnofsky. Significant differences between groups were indicated when p < 0.05.

## Data Availability

Data collected for the study, including individual participant data and a data dictionary defining each field in the set, will be made available to others; deidentified participant data, participant data with identifiers, data dictionary, or other specified data set will be made available; study protocol, statistical analysis plan, informed consent form, and other related documents will be available; with publication, these data will be available; these data can be obtained by contacting the corresponding author Xiaodong Ma at the email address xiaodongm@hotmail.com ; with investigator support, after approval of a proposal and with a signed data access agreement, data will be shared.
